# A Top-Down Approach and Thermal Characterization of Luminescent Hybrid BPA.DA-MMA@Ln_2_L_3_ Materials Based on Lanthanide(III) 1H-Pyrazole-3,5-Dicarboxylates

**DOI:** 10.3390/ma15248826

**Published:** 2022-12-10

**Authors:** Renata Łyszczek, Dmytro Vlasyuk, Beata Podkościelna, Halina Głuchowska, Ryszard Piramidowicz, Anna Jusza

**Affiliations:** 1 Department of General and Coordination Chemistry and Crystallography, Institute of Chemical Sciences, Faculty of Chemistry, Maria Curie–Skłodowska University, M. Curie-Skłodowskiej Sq. 2, 20-031 Lublin, Poland; 2 Department of Polymer Chemistry, Institute of Chemical Sciences, Faculty of Chemistry, Maria Curie–Skłodowska University, Gliniana 33, 20-614 Lublin, Poland; 3 Institute of Microelectronics and Optoelectronics, Warsaw University of Technology, Koszykowa 75, 00-662 Warsaw, Poland

**Keywords:** 1H-pyrazole-3,5-dicarboxylates, polymers, hybrid materials, luminescence, thermal analysis

## Abstract

In this study, novel hybrid materials exhibiting luminescent properties were prepared and characterized. A top-down approach obtained a series of polymeric materials with incorporated different amounts (0.1; 0.2; 0.5; 1, and 2 wt.%) of dopants, i.e., europium(III) and terbium(III) 1H-pyrazole-3,5-dicarboxylates, as luminescent sources. Methyl methacrylate and bisphenol A diacrylate monomers were applied for matrix formation. The resulting materials were characterized using Fourier transform infrared spectroscopy (FTIR) and thermal analysis methods (TG-DTG-DSC, TG-FTIR) in air and nitrogen atmosphere, as well as by luminescence spectroscopy. The homogeneity of the resulting materials was investigated by means of optical microscopy. All obtained materials exhibited good thermal stability in both oxidizing and inert atmospheres. The addition of lanthanide(III) complexes slightly changed the thermal decomposition pathways. The main volatile products of materials pyrolysis are carbon oxides, water, methyl methacrylic acid and its derivatives, bisphenol A, 4-propylphenol, and methane. The luminescence properties of the lanthanide complexes and the prepared hybrid materials were investigated in detail.

## 1. Introduction

Intensive development of luminescent hybrid materials has been observed in recent years, due to their huge potential applications in many fields including optoelectronics, laser systems, chemical sensors, optical communication devices, protection coating, etc. [1,2,3,4,5,6,7,8,9,10]. Hybrid materials are typically composed of at least two different components connected at the nano-metric or molecular level, where one of the components plays the matrix role [3,11,12]. These components introduce different properties, thus the final advanced materials exhibit improved and desirable features. This approach allows the generation of structurally diverse materials designed for special applications. Many hybrid materials are based on polymer matrices, due to their easy processing, mechanical resistance, low density, good flexibility, and controllable cost. Polymers such as poly(methyl methacrylate), poly(vinyl alcohol), polyethylene, polycarboxylate, poly(divinylbenzene), and others have been well examined as matrices [13,14,15,16,17]. On the contrary, the polymerization of Bisphenol A diacrylate (BPA.DA) and methyl methacrylate (MMA) monomers generates a polymer that has been comparatively neglected as a matrix in the production of hybrid materials, despite its wide use in various fields of science and industry, including packaging and coating materials, due to its high transparency, heat-deflection temperature, and impact strength, good ultraviolet stability, fire resistance, and excellent mechanical properties [18,19,20,21,22,23,24]. These desirable features arise from the fact that aromatic BPA.DA plays a crucial crosslinking role during the polymerization of these monomers, making this polymer an excellent matrix for the formation of hybrid materials containing dopants insoluble in monomers.

The luminescent properties of hybrid materials can originate from different sources, such as the matrix, additives at small concentrations, or resulting from the synergistic effects of the properties of the parent components. Among numerous compounds, the selected lanthanide complexes are perfect candidates for luminescent dopants, owing to the unique optical properties of Ln(III) ions. Their emission spectra show narrow bands derived from the f-f transitions in the visible (VIS) and near-infrared (NIR) ranges, but their intensities are low because lanthanide ions suffer from weak light absorption [3,25,26]. Many lanthanide complexes with organic ligands exhibit intense luminescence, because of the effective intramolecular energy transfer from the coordinated ligands to the luminescent central lanthanide ions. In such cases, the lanthanide complexes exhibit sensitized luminescence known also as the “antenna effect” [3,25,26,27,28,29,30].

The earliest reported luminescent hybrid materials were doped with molecular lanthanide complexes constructed predominantly of chelating agents [3,31,32,33]. Despite the preferable luminescent properties of such complexes and their good solubility, many of them suffer from low thermal stability that strongly impacts their final material properties. One possible way of overcoming such a disadvantage is to use more sustainable compounds as dopants. Carboxylate lanthanide complexes are considered to be thermally stable due to the formation of strong Ln-O bonds. In many of these complexes, organic ligands act as a light-harvesting system participating in the indirect sensitization of Ln(III) ions, which has led to the frequent application of these compounds as luminescence origins in hybrid materials [8,34,35,36]. In previously reported luminescent hybrid materials based on polymeric matrices, lanthanide carboxylates of molecular and polymeric structures were used as dopants [35,36].

Thermal analysis methods play a significant role in the characterization of novel materials, including luminescent hybrid materials for a variety of possible applications. Studies of the thermal behavior of different classes of chemicals and organic, inorganic, and hybrid materials should be conducted to assess their thermal stability and to verify their potential to cause accidents, fire, or environmental pollution [37,38]. Therefore, these studies are essential for supporting theoretical models with practical purposes. Furthermore, many new or existing materials can undergo thermal degradation, combustion, or pyrolysis (depending on the reaction temperature and the degree of oxygen consumption), and the results obtained can be used to draw appropriate conclusions on a possible recycling procedure for energy recovery and and/or production of valuable oily chemical substances. In common practice, studies of the thermal behavior of materials are considered a preliminary test measure to discover the most appropriate temperature ranges at which pre-treatment can be carried out to elicit a given property for an required application [39,40]. Materials and composites including antibiotics, polyhedral oligomeric silsesquioxane nanocomposites polymers, and organic–inorganic hybrids are frequently tested by experimental thermogravimetric-differential scanning calorimetry (TG-DSC). Data are obtained for kinetic parameters related to thermal decomposition reactions, in order to evaluate their thermal stability [41].

This article is a continuation of our research on the synthesis and characterization of hybrid materials formed by the incorporation of newly prepared lanthanide(III) complexes bearing desired functional features, e.g., luminescence, into the polymeric matrices [35,36]. Such a procedure in the case of the formation of hybrid materials containing coordination polymers is regarded as a top-down approach. In contrast, generation of metal complexes along with target hybrid materials is referred to as a bottom-up procedure [16].

The purpose of this paper is to present the top-down approach to synthesizing new hybrid materials from BPA.DA-MMA, a rarely used polymer matrix, and lanthanide(III) complexes of 1H-pyrazole-3,5-dicarboxylic acid such as [Eu_2_(Hpdca)_3_(H_2_O)_6_] and [Tb_2_(Hpdca)_3_(H_2_O)_6_]. The selected organic ligand forms with lanthanides(III) coordination polymers (LnCPs) because of the diverse coordinating modes and variabilities of deprotonation of the carboxylic groups [42,43]. The most recent report on LnCPs constructed from H_3_pdca acid focused mainly on their synthesis methods, crystal structures, and thermal properties [43]. However, investigations into the potential applications of such lanthanide complexes, including formation of luminescent hybrid materials, have not yet been reported. Therefore, we aimed to determine whether the above lanthanide complexes would function as luminescent additives in the prepared materials.

Incorporation of lanthanide(III) coordination polymers at different concentrations of 0.1, 0.2, 0.5, 1, and 2 wt.% into the BPA.DA-MMA matrix led to the formation of materials which possessed both the characteristic luminescence of lanthanide(III) ions and the attractive features of organic polymers, including mechanical strength, flexibility, transparency, and ease of processing. A principal feature that determines the potential application of the materials is their thermal stability. Therefore, particular attention was paid to the examination of the thermal behaviour of the obtained materials. The pathways of thermal decomposition in the reported materials were examined in air using the thermogravimetric (TG) and differential scanning calorimetry (DSC) methods. The thermal degradation of the materials was also evaluated by means of the evolved thermal analysis method incorporating the TG-FTIR technique, allowing the mass loss and FTIR spectra of the evolved compounds to be recorded during the samples’ heating in an air and nitrogen atmosphere. The impact of the processing on the pathways of thermal decomposition in the polymeric matrix and hybrid materials in an oxidizing atmosphere was also evaluated. The luminescence properties of the [Eu_2_(Hpdca)_3_(H_2_O)_6_] and [Tb_2_(Hpdca)_3_(H_2_O)_6_] complexes and those of the obtained hybrid materials were furthermore investigated. The role of 1H-pyrazole-3,5-dicarboxylate ligand in the enhancement of lanthanide-centered emission ions was established.

## 2. Materials and Methods

Bisphenol A glycerolate (1 glycerol/phenol) diacrylate (BPA.DA), methyl methacrylate (MMA), and 2,2-dimethoxy-2-phenylacetophenone (Irgacure 651, IQ) were purchased from Sigma-Aldrich, (Darmstadt, Germany). All the chemical reagents and materials were obtained from commercial sources and used without further purification.

The lanthanide complexes [Eu_2_(Hpdca)_3_(H_2_O)_6_] and [Tb_2_(Hpdca)_3_(H_2_O)_6_] were prepared using procedures previously described in the reaction of lanthanide(III) salt with sodium salt of 1H-pyrazole-3,5-dicarboxylic acid under hydrothermal conditions [43].

### 2.1. Synthesis of Hybrid Materials

MMA and BPA.DA monomers were mixed in proportions of 70:30 wt.%, and then Irgacure 651 was added at 1 wt.% as the photoinitiator. Next, the solid [Eu_2_(Hpdca)_3_(H_2_O)_6_] complex was added to the obtained mixture in proportions of 0.1, 0.2, 0.5, 1, and 2 wt.%. The prepared mixtures were heated at 80 °C to remove air bubbles. The well-homogenized mixtures were poured into glass molds (10 × 12 × 0.2 cm) with a Teflon spacer and exposed to UV radiation (8 lamps, each 40 W). The UV polymerization time was 25–30 min, then the resulting composites were heated at 80 °C for 2 h for final cross-linking. The free matrix and the hybrid materials were obtained in the form of blocks.

### 2.2. Instrumentation and Methods

The attenuated total reflectance Fourier transform infrared spectra (ATR-FTIR) of the free matrix and the prepared hybrid materials were recorded using a Nicolet 6700 spectrophotometer equipped with a Smart iTR accessory (diamond crystal) over the range 4000–600 cm^−1^.

The transmittance FTIR spectra of pure matrix, BPA.DA-MMA@2%Eu_2_L_3_, and BPA.DA-MMA@2%Tb_2_L_3_ materials were collected using a Jasco FT/IR-4600LE spectrophotometer in the range 4000–400 cm^−1^. Thermal analyses of the synthesized materials were carried out, applying the thermogravimetric (TG) and differential scanning calorimetry (DSC) methods using a SETSYS 16/18 analyzer (Setaram). The samples (mass about 5–9 mg) were heated in alumina crucibles up to 1000 °C at a heating rate of 10 °C min^−1^ in a dynamic air atmosphere (12.5 cm^3^ min^−1^). The TG curves and the FTIR spectra of the evolved gases from the investigated samples were recorded using a Q5000 thermal analyzer (TA Instruments) coupled with a Nicolet 6700 spectrophotometer. Samples of 20–30 mg were heated in a dynamic nitrogen and air atmosphere (25 cm^3^ min^−1^) at a heating rate of 10 and 20 °C min^−1^. The samples were heated to 700 °C in open platinum crucibles. The transfer line was heated up to 250 °C and the gas cell of the spectrophotometer was heated to 240 °C.

The fragments of the solid composites were studied using a Morphologi G3 optical microscope (Malvern, UK). The scanning electron microscopy (SEM) images were recorded with a Quanta 3D FEG (FEI).

The excitation spectra, emission spectra, and profiles of fluorescence decay were recorded using a Horiba PTI QuantaMasterbased modular spectrofluorimetric system equipped with double monochromators in the excitation and emission paths, enabling both CW and pulsed excitations (Xe CW lamp of 75 W maximum power, Xe pulsed lamp of maximum frequency 300 Hz, pulse duration 1 μs) over a wide spectral range (200–2000 nm). All measurements were taken at room temperature, and all spectra were corrected for the spectral characteristics of the detector’s response.

## 3. Results and Discussion

### 3.1. Morphological Analysis

**Figure 1:**
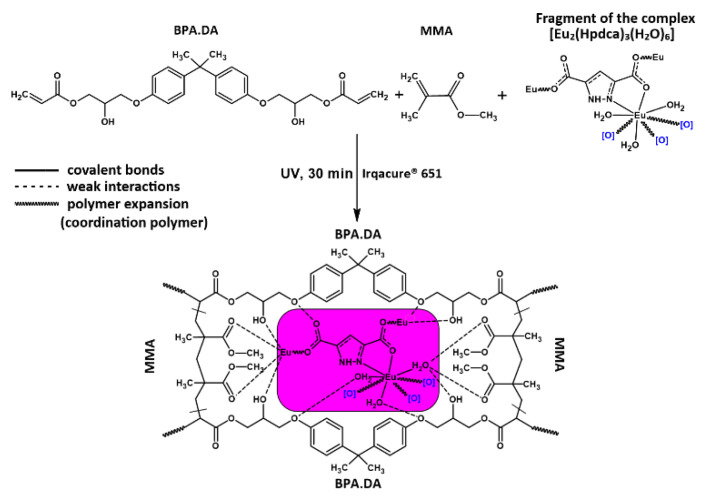
Scheme of the hybrid materials’ synthesis and proposed structure of obtained materials based on the europim(III) complex.

A series of hybrid materials BPA.DA-MMA@Ln_2_L_3_ composed of the cross-linked polymeric matrix BPA.DA-MMA (BPA.DA-bisphenol A diacrylate and MMA-methyl methacrylate monomers) and europium(III) and terbium(III) complexes of 1H-pyrazole-3,5-dicarboxylic acid (Ln_2_L_3_: Eu_2_L_3_ = [Eu_2_(Hpdca)_3_(H_2_O)_6_] or Tb_2_L_3_ = [Tb_2_(Hpdca)_3_(H_2_O)_6_]) were synthesized and characterized. The polymeric matrix and hybrid materials containing different amounts of dopants, i.e., lanthanide complexes (Table S1), were prepared using the ultraviolet polymerization procedure. The scheme of the hybrid materials’ synthesis and proposed structure of the materials is presented in Figure 1.

**Figure 2:**
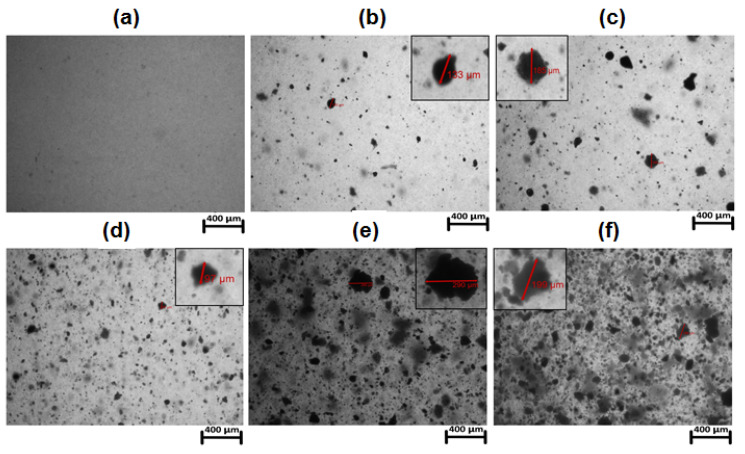
Optical microscope images: (**a**) BPA.DA-MMA; (**b**) BPA.DA-MMA@0.1%Eu_2_L_3_; (**c**) BPA.DA-MMA@0.2%Eu_2_L_3_; (**d**) BPA.DA-MMA@0.5%Eu_2_L_3_; (**e**) BPA.DA-MMA@1%Eu_2_L_3_; (**f**) BPA.DA-MMA@2%Eu_2_L_3_.

**Figure 3:**
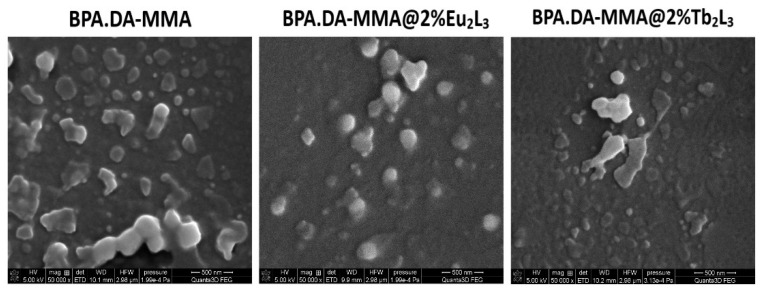
SEM images (1 μm magnification) of free matrix and hybrid materials doped with 2 wt.% of Eu_2_L_3_ and 2 wt.% of Tb_2_L_3_.

The polymeric matrix was transparent, and the hybrid materials became cloudy with the increasing content of lanthanide dopants. Microscopic examination showed that in most cases the particles of the incorporated lanthanide complexes of irregular shapes had diameters in the range of 10–290 μm (Figure 2). The scanning electron microscope (SEM) images of the block pure matrix and hybrid materials are shown in Figure 3. The materials’ surfaces are not smooth and irregular shaped particles can be distinguished.

### 3.2. Infrared Spectroscopy Analysis

The ATR-FTIR spectra of the pure matrix (BPA.DA-MMA) and the hybrid materials were strongly dominated by the bands derived from the polymeric matrix BPA.DA-MMA (Figures S1 and S2). The stretching vibrations of the C_Ar_H and OH groups from bisphenol A moieties and the stretching vibrations of CH from the aliphatic chains appeared in the wavenumber range 3600–3100 cm^−1^. The FTIR spectra exhibitedbands at 2950 and 2875 cm^−1^ originating from the stretching vibrations of the CH_2_ and CH_3_ groups (Figures S1 and S2). The bands of the deformation vibrations in those groups occurred at 1455 and 1385 cm^−1^. The intense band at 1726 cm^−1^ is attributed to the stretching vibrations of the carbonyl groups ν(C=O) from the polymer ester parts. Several peaks at 1634, 1607, 1581 and 1508 cm^−1^ resulted from the stretching vibrations of ν(C_Ar_C_Ar_) from the aromatic rings. The intense and broad bands at 1236 cm^−1^ and 1180 cm^−1^ were assigned to the stretching vibrations of the ν(C-O) and ν(C-O-C) groups from ester moieties. Several bands in the region 1060–600 cm^−1^ at wavenumbers 1039, 1011, 984, 828, 758, 737, and 727 cm^−1^ can be assigned to the skeletal stretching modes of ν(CC) of methyl(methacrylate) moieties, as well as γ(C_Ar_C_Ar_), γ(C_Ar_H) out-of-plane bending and the torsion motions of aromatic rings [24,44,45,46,47].

Incorporation of small amounts of dopants did not strongly affect the ATR-FTIR spectra of hybrid materials. The characteristic bands of lanthanide(III) complexes overlapped with those of the polymeric matrix. The only differences among the infrared spectra of the pure matrix and the obtained materials were observed on the transmittance FTIR spectra (Figure S3). The infrared spectrum of the pure matrix exhibited a very strong diagnostic band at 1732 cm^−1^ due to ν(C=O), which shifted slightly towards the lower wavenumbers 1728 cm^−1^ in the spectra of BPA.DA-MMA@2%Ln_2_L_3_ materials [18,35,36]. This observation can confirm the participation of functional groups in binding metal centers of the embedded lanthanide complexes, formation of hydrogen bonds, and/or the presence of other kinds of weak interactions between the components of the hybrid materials. The addition of a small amount of the lanthanide complexes did not cause visible changes in the infrared spectra of the obtained materials, which may be the result of the low intensity of the characteristic vibrations of the dopants. Therefore, the bands in the ranges 1597–1579 and 1359–1348 cm^−1^ derived from the asymmetric and symmetric stretching vibrations of carboxylate groups (-COO) characteristic for lanthanide complexes were obscured by the matrix bands in the ranges 1607–1581 cm^−1^ [43]. Furthermore, the bands from skeletal stretching vibrations (C_Ar_C_Ar_) of metal complexes overlapped with corresponding bands originated from the skeletal vibration of the polymeric matrix.

### 3.3. TG-DSC Analysis of BPA-MMA Matrix and Hybrid Materials

**Figure 4:**
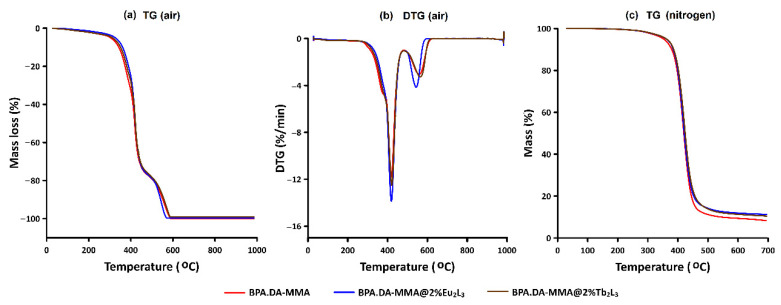
(**a**) TG and (**b**) DTG curves of the BPA.DA-MMA matrix, BPA.DA-MMA@2%Eu_2_L_3_, and BPA.DA-MMA@2%Tb_2_L_3_ materials (block form) in air atmosphere. (**c**) TG curves of materials (block form) in nitrogen atmosphere.

The TG curves of the BPA.DA-MMA matrix and the synthesized hybrid materials had similar shapes under the same measurement conditions (Figure 4 and Figures S4–S7). However, a more careful analysis of the recorded TG curves revealed certain subtle differences due to the presence of lanthanide dopants in the investigated hybrid materials (Table 1 and Table 2). The additives were found to affect the thermal stability of hybrid materials as well as the mechanism of their decomposition. The influencing effects of atmosphere temperature and materials’ processing on their thermal stability and pathways of decomposition were also evaluated.

The thermal data are presented for the as-synthesized materials, i.e., in the form of blocks in an air atmosphere. Particular attention is drawn to the materials with 2% addition of dopants, for which the incorporated additives had the strongest impact on the material properties. 

Taking into account the shape of the TG-DTG curves of the matrix and synthesized materials BPA.DA-MMA@Eu_2_L_3_ and BPA.DA-MMA@Tb_2_L_3_ in an air atmosphere, two main stages of decomposition were distinguished (Figure 4 and Figures S4–S7, Table 1 and Table 2). Mass loss of about 74% was recorded for the matrix and materials doped with 2% lanthanide complex in the temperature ranges 229–448, 163–454, and 119–454 °C, respectively.

As can be seen from the TG and DTG curves of the matrix and hybrid materials (Figure 4 and Figures S4–S7), continuous mass losses (in the first stage) up to 311–340 °C connected with the release of humidity and/or water molecules from hydrated lanthanide complexes acting as dopants. The dehydration process in lanthanide(III) 1H-pyrazole-3,5-dicarboxylates occurred up to about 200 °C (Figure S8) [43]. For the pure matrix, mass losses of 1, 5, 20, and 50% on the TG curve were recorded at 228, 340, 399 and 420 °C, respectively. According to the thermal data corresponding to the first stage, mass losses in the materials of 1, 5, and 20% were found at lower temperatures compared to the free matrix (air). Mass losses of 50% in these materials were recorded at temperatures close to that observed for the matrix (Table 2).

At higher (air) temperatures, the decomposition process was reflected by significant mass changes recorded on the TG curves. The polymeric matrix displayed the highest thermal stability. The data presented in Table 2 indicate that the material doped with terbium(III) complex had lower thermal stability in comparison with corresponding materials containing the europium(III) dopant. The material with 2% addition of Tb_2_L_3_ complex exhibited the lowest stability. The observed slight differences in the thermal behaviors of the pristine matrix and hybrid materials are related to the thermal properties of the embedded lanthanide complexes [43] and their interaction with polymer framework. The discrepancies among corresponding hybrid materials containing the same percentage of metal complex may be related to the different thermal decomposition of metal complexes above 300 °C (Figure S8).

As follows from the DSC curves (in air), the first stage was dominated by endothermic effects driven by the overlapping melting and decomposition processes of polymeric matrix as well as the dopants’ decomposition (Table 1, Figures S6 and S7). The top peaks of the first endothermic effects for the free matrix and hybrid materials with 2% added lanthanide complex were observed at very similar temperatures (401.2, 401.2, and 389.7 °C), while the observed values of endothermic effects differed. The lowest endothermic effect (11.3 J/g) was recorded for pure BPA.DA-MMA material. The presence of dopants caused an increase of endothermic effect values to 512.6 and 220.3 J/g for BPA.DA-MMA@2%Eu_2_L_3_ and BPA.DA-MMA@2%Tb_2_L_3_, respectively. At a slightly higher temperature, endothermic effects (129.8, 77.9, and 343.8 J/g) were observed in the temperature range 414–448 °C (peak) for the BPA.DA-MMA and hybrid materials. Regarding BPA.DA-MMA@2%Tb_2_L_3_, a third endothermic effect at 440.6 °C with ∆H of 130.5 J/g was observed (Table 1). The existence of these endothermic effects can be explained in terms of the pathway of the decomposition process, as well as the thermal properties of solid products’ decomposition. The recorded difference in the energy of the endothermic effects may indicate diverse interactions between the matrix and the lanthanide complex incorporated in the materials. The higher value of the endothermic effects for BPA.DA-MMA@2%Eu_2_L_3_ in comparison with BPA.DA-MMA@2%Tb_2_L_3_ indicates stronger interactions between the europium complex and the polymeric matrix (Figure 1) due to the possible formation of hydrogen bonds and Van der Waals interactions [3].

Further heating in air resulted in the decomposition of unstable intermediate products, observed in the temperature ranges 449–592, 455–586, and 455–607 °C for the matrix and the BPA-MMA@2%Eu_2_L_3_ and BPA-MMA@2%Tb_2_L_3_ materials, respectively. The observed mass losses in the range of 25.2–26.7% (Table 1) were accompanied by very strong exothermic effects on the DSC curves due to the burning of organic solid residues (Figures S5–S7). Heating of the matrix in air resulted in a lack of any solid residues at 1000 °C, while for the investigated hybrid materials only traces (below 0.5%) of corresponding lanthanide oxides, i.e., Eu_2_O_3_ or Tb_4_O_7_, were noticed.

The impact of atmosphere temperature on the thermal stability of the block materials under investigation was determined by heating selected materials (BPA.DA-MMA, BPA.DA-MMA@2%Eu_2_L_3_, and BPA.DA-MMA@2%Tb_2_L_3_) in nitrogen. The profiles of the recorded TG curves were similar (Figure 4c). All materials exhibited higher thermal stability in nitrogen in comparison with the air atmosphere (Table S2) where additional oxidation reactions take place alongside the decomposition process [48]. Mass loss of 1% was recorded for the tested materials in the range 261–267 °C. The comparative thermal stabilities of the matrix and the investigated hybrid materials were very similar. At higher temperatures, significant mass loss of about 85% was observed due to the pyrolysis process. This stage took place in the temperature range 262–500 °C. Further heating resulted in slight changes of mass, recorded on the TG curves, and final solid residues were observed at 700 °C. The total mass loss for the free matrix was 91.8%, while for the BPA.DA-MMA@2%Eu_2_L_3_ and BPA.DA-MMA@2%Tb_2_L_3_ materials the corresponding final mass losses were 89.0% and 89.5%, respectively. In the case of solid residues of hybrid materials, in addition to unburnt carbon (free matrix), lanthanide oxides were also formed [18,35,36,38].

In addition to thermal analysis of the as-synthesized materials in the form of blocks, the powdered materials were also investigated. The profiles of the TG and DSC curves in air indicate clearly their different thermal stabilities and mechanisms of decomposition (Figures S4–S7). In general, the thermal stability of all the powdered materials was reduced compared with that of the blocks. Changes in the temperature associated with losses of 1, 5, 20, and 50% mass in comparison with block hybrid materials were in the ranges 3–50, 20–36, 34–55, and 43–48 °C, respectively. Temperatures resulting in 1% mass loss for the series of powdered BPA.DA-MMA@Eu_2_L_3_ materials were decreased by 34, 38, 28, 26, and 3 °C in comparison to corresponding block materials. For the series of powdered BPA.DA-MMA@Tb_2_L_3_ materials, temperatures for 1% mass loss were lowered by 8, 9, 23, 50, and 37 °C compared with block materials. The temperatures corresponding to 5, 20, and 50 % mass loss were similar for the respective powdered hybrid materials in the two series. The temperature associated with 1% of mass loss for powdered BPA-MMA decreased up to 121 °C (228 °C for the block), while for other analyzed points on the TG curves, the temperature alteration was not so drastic. It can be stated that the powdered hybrid materials containing lanthanide complexes did not demonstrate such a dramatic loss of stability relative to the powder matrix.

### 3.4. TG-FTIR Analysis of BPA-MMA Matrix and Hybrid Materials

**Figure 5:**
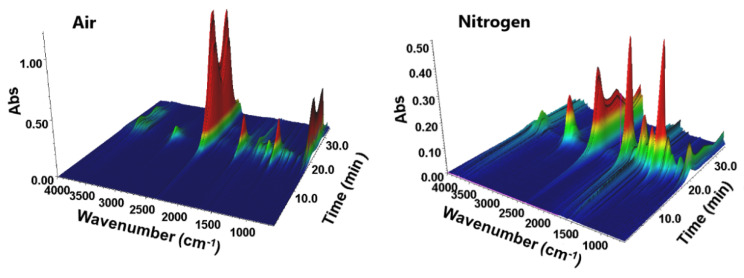
3D projection of the FTIR spectra of volatile products of BPA.DA-MMA@2%Eu_2_L_3_ material decomposition in air and nitrogen atmosphere.

Thermogravimetric measurements (TG) were taken and spectroscopic identification (FTIR) performed of the evolved gases in nitrogen and air for the matrix and materials (block form) with the highest concentration of dopant, i.e., 2 wt.%. The Gram–Schmidt plots representing the total intensities of all evolved gases as a function of time (temperature) during heating of the materials in nitrogen are provided in Figure S9. As a representative example, the 3D FTIR spectra of the volatile products of BPA.DA-MMA@2%Eu_2_L_3_ material decomposition are shown in Figure 5.

The FTIR spectra of volatile products of the investigated materials after heating in nitrogen indicated that water molecules evolved first. Diagnostic bands of weak intensity in the wavenumber ranges 4000–3500 and 1800–1300 cm^−1^ were due to the stretching and deformation vibrations of OH groups of evolved water molecules, and were observed up to about 270 °C. Next, further compounds such as carbon oxides and various organic compounds were released as products of the materials’ degradation in nitrogen.

**Figure 6:**
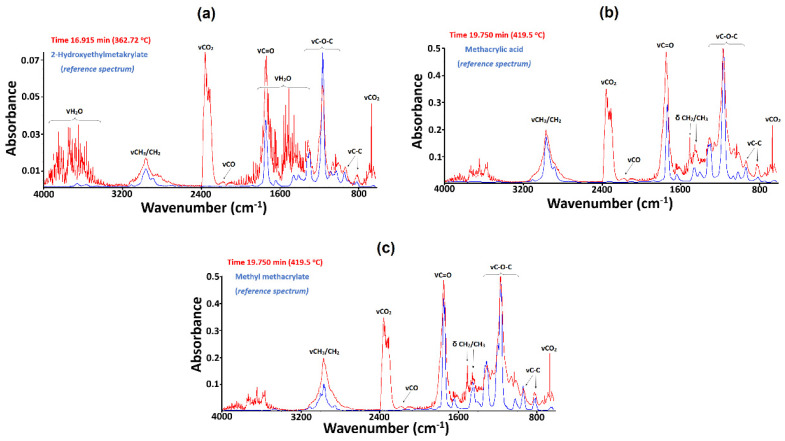
Experimental infrared spectra of gaseous products recorded in the nitrogen atmosphere at different temperatures are indicated in red, alongside FTIR spectra of references represented in blue: (**a**) 2-hydroxyethylmethacrylate, (**b**) methacrylic acid, and (**c**) methyl methacrylate.

The infrared spectra show strong diagnostic bands with maxima at 2359 and 2343 cm^−1^ and others in the range 750–600 cm^−1^ ascribed to the stretching and deformation vibrations of carbon(IV) oxide molecules. The diagnostic double band with maxima at 2185 and 2107 cm^−1^ corresponds to the stretching vibrations of carbon oxide(II) [49]. Methacrylic acid and its derivatives such as methyl methacrylate and 2-hydroxyethylmethacrylate (Figure 6) are the main products of bonds breaking in the polymeric matrix [18,24,35,36].

The broad band in the range 3150–2800 cm^−1^ centered at 2972 cm^−1^ was attributed to the stretching vibrations of =CH_2_ and -CH_3_ moieties. The very intense band at 1748 cm^−1^ can be assigned to the stretching vibrations of carbonyl groups from the carboxylic and ester groups. Deformation vibrations of the CH_2_/CH_3_ groups were found at 1456 and 1362 cm^−1^. The band at 1306 cm^−1^ and those at 1166 cm^−1^, 1061, and 1031 cm^−1^ were found to correspond to the stretching vibrations of C-O-C ester groups. The absorption bands at 936 and 813 cm^−1^ are derived from the skeletal vibrations of C-C from the aliphatic moieties [44,45,46,47]. Figure 6 presents the experimental and reference FTIR spectra of identified compounds recorded at 362.72 and 419.50 °C. Further heating of the materials above 420 °C also resulted in the evolution of gaseous 4,4′-(propane-2,2-diyl) diphenol (bisphenol A, BPA) and its decomposition products such as methane and 4-propylphenol [50,51,52,53].

**Figure 7:**
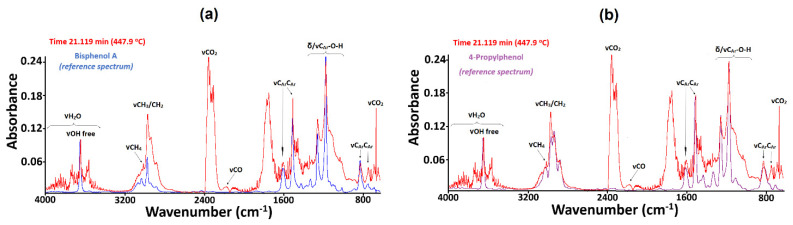
Experimental infrared spectra of gaseous products (shown in red) recorded at different atmosphere, along with FTIR spectra of references (shown in blue): (**a**) bisphenol A (air); (**b**) 4-propylphenol (nitrogen).

Alongside the bands from methacrylate compounds, water, and carbon oxides, the FTIR spectrum recorded at 447 °C displays characteristic diagnostic vibrations from bisphenol A and its decomposition compounds (Figure 7). The vibrations from the free hydroxyl groups of BPA produced a relatively strong band at 3649 cm^−1^. The presence of two methyl groups in the released molecules of BPA is reflected in the stretching and deformation vibrations found at 2972, 1472, and 1362 cm^−1^. The bands at 1602 and 1507 cm^−1^ and those at 826 and 746 cm^−1^ are derived from the stretching and out-of-plane deformation vibrations of C_Ar_C_Ar_ and C_Ar_H bonds from the aromatic rings. Additionally, the FTIR spectra were found to exhibit very intense double bands with maxima at 1258 and 1174 cm^−1^ assigned to the C_Ar_-O-H groups from the phenol moieties. Methane molecules as the product of BPA molecules’ degradation showed significant diagnostic bands in the range 3200–2950 cm^−1^ with a characteristic maximum at 3015 cm^−1^ due to the stretching vibrations of CH bonds. Evidence of 4-propylphenol formation as the bisphenol A decomposition product is reflected in the presence of the band at 2937 cm^−1^ from the stretching vibrations of CH_2_ groups within the propyl substituent [51,52,53,54]. The reference spectrum of the compound fits well the experimental one.

The FTIR spectra of the gaseous decomposition products of the pure matrix and the BPA.DA-MMA@2%Eu_2_L_3_ and BPA.DA-MMA@2%Tb_2_L_3_ materials after heating in air were also recorded. The profiles of the TG curve were very similar to those in the air atmosphere described above. The differences observed were associated with the diverse measurement conditions such as heating rate and rate of air flow. The TG curves’ profiles confirm the two-stage decomposition process (Figure S4). The first significant mass change took place up to about 448 °C, with mass losses of 80.7 and 78.1% for the matrix and the hybrid materials, respectively. In this step, similarly to the observations under the nitrogen atmosphere, the FTIR spectra of evolved gases were dominated by characteristic bands from methacrylate derivatives, methane, and carbon oxides (Figure S10). It is worth mentioning that bisphenol A molecules were liberated in the inert atmosphere. A second mass loss of about 20% indicated by the TG occurred in the temperature range 450–600 °C. The recorded FTIR spectra show very strong bands from the carbon oxides while the bands from hydrocarbons are almost invisible. These gaseous products confirm that oxidizing processes of unstable solid residues occur mainly above 450 °C. For the investigated matrix, almost 100% mass loss was observed at 700 °C. Heating the hybrid materials resulted in 0.5% mass of residue due to the formation of suitable oxides (Eu_2_O_3_, Tb_4_O_7_).

### 3.5. Luminescent Properties of Lanthanide Complexes and Powdered Hybrid Materials

For all investigated samples, the luminescent properties were also analyzed in the short-wavelength spectral range and are discussed here. Due to the specific (powdered) type of original active media and the plates’ lack of transparency, the typical measurements of the absorption characteristics were replaced by excitation spectra measurements. For all concentrations of Tb(III) and Eu(III) complexes in the hybrid materials the emission and excitation properties remained the same, differing only in the signal intensities, therefore only the results for the highest concentrations are presented.

**Figure 8:**
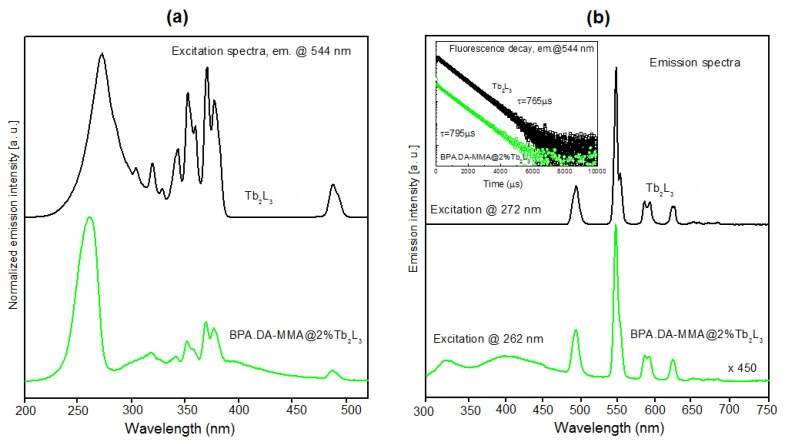
(**a**) Comparison of the excitation characteristics recorded for the Tb_2_L_3_ complex and the BPA.DA-MMA@2%Tb_2_L_3_ hybrid material (544 nm emission). (**b**) Comparison of the emission characteristics recorded for the Tb_2_L_3_ complex and the BPA.DA-MMA@2%Tb_2_L_3_ hybrid material (UV excitation).

**Figure 9:**
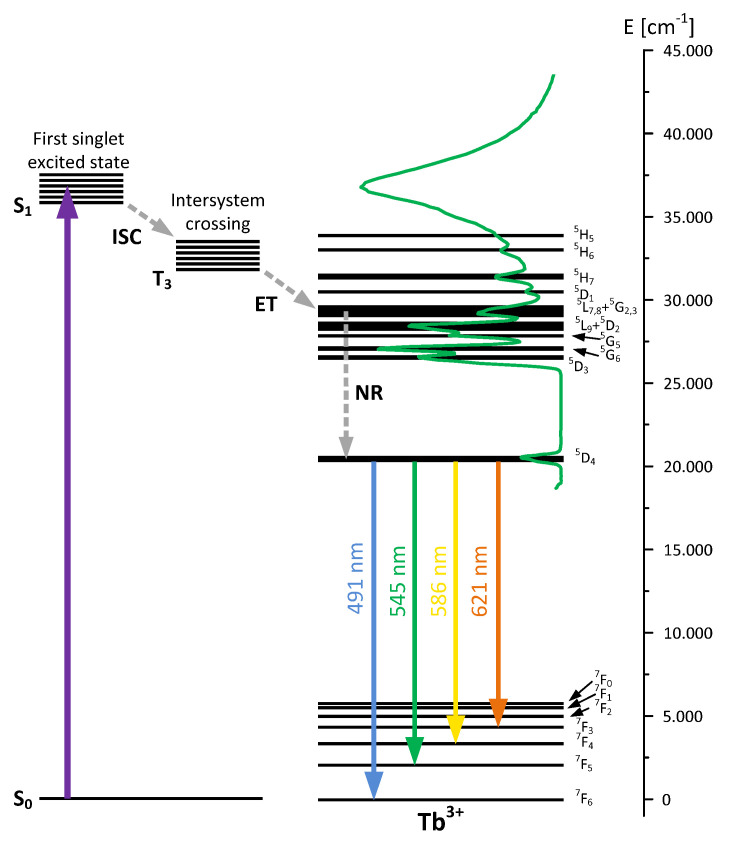
Energy transfer mechanism for Tb(III) sensitized fluorescence as most efficient excitation path together with excitation spectrum and emission transitions from ^5^D_4_ level (ISC—intersystem crossing, ET—energy transfer, NR—non-radiative transitions).

Figure 8a shows a comparison of the excitation spectra of Tb_2_L_3_ complex and BPA.DA-MMA@2%Tb_2_L_3_ hybrid material recorded for the most intense green emission (544 nm). The strong broadband absorption bands in the UV region correspond to the most efficient excitation of terbium(III) ions via ligands (energy transfer from the triplet state of the ligand, preceded by absorption to the singlet state and intersystem crossing ISC to the triplet state, as shown in detail in Figure 9) [3,25,55,56]. Narrow lines related to the direct excitation of high-position energy levels of terbium(III) are also clearly visible. The recorded spectra for the complex and hybrid materials differed mainly in the position of maximal intensity of absorption through the ligand–ion energy transfer (which shifted towards shorter wavelengths for the composites).

On the luminescence spectra (Figure 8b), several emission lines typical of terbium(III) activated materials are clearly observable for both the complex and the composite material. The most intensive (centered at 544 nm) is evidently related to ^5^D_4_ → ^7^F_5_ transition while the remaining four lines correspond to ^5^D_4_ → ^7^F_6,4,3,2_ transitions [30,55,56] (shown schematically in Figure 9). The luminescence spectrum recorded for hybrid material differed only slightly from that observed for the terbium(III) complex. The spectral positions of all lines were the same, but the lines were slightly wider and the parasitic broadband luminescence extended from 290 nm to 470 nm, which was clearly observable and could be attributed to emission from the polymeric host. The luminescence decay profiles (shown in the insert) had the same character for all observable lines and were purely exponential, with a considerably lengthy time constant of 750–800 µs for both the complex and the hybrid materials.

**Figure 10:**
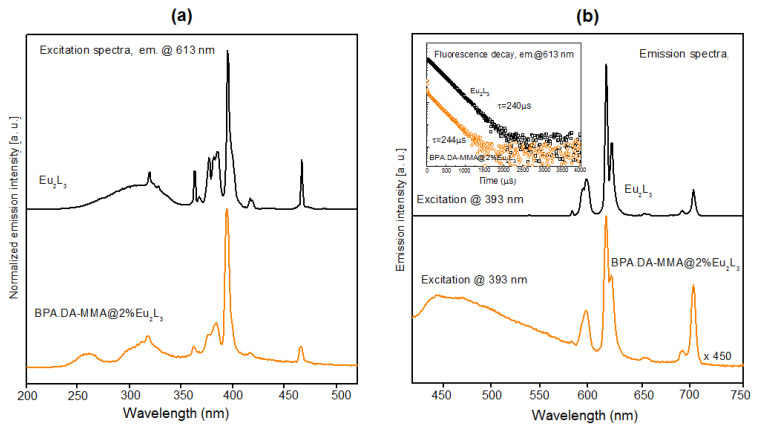
(**a**) Comparison of the excitation characteristics recorded for the Eu_2_L_3_ complex and BPA.DA-MMA@2%Eu_2_L_3_ composite material (recorded for 613 nm emission). (**b**) Comparison of emission characteristics recorded for the Eu_2_L_3_ complex and BPA.DA-MMA@2%Eu_2_L_3_ hybrid material (393 nm excitation).

In the case of Eu_2_L_3_ doped materials, in the excitation spectra recorded for all samples (Figure 10a) the ligand–ion energy transfer band was suppressed and the optimal mechanism of exciting europium ions was the direct excitation of Eu levels (the “antenna effect” being insufficient). This means that this particular ligand is not a good sensitizer for Eu(III) luminescence. For hybrid materials, the additional excitation band visible in the UV range (ca. 260 nm) is probably related to the polymer host [57].

**Figure 11:**
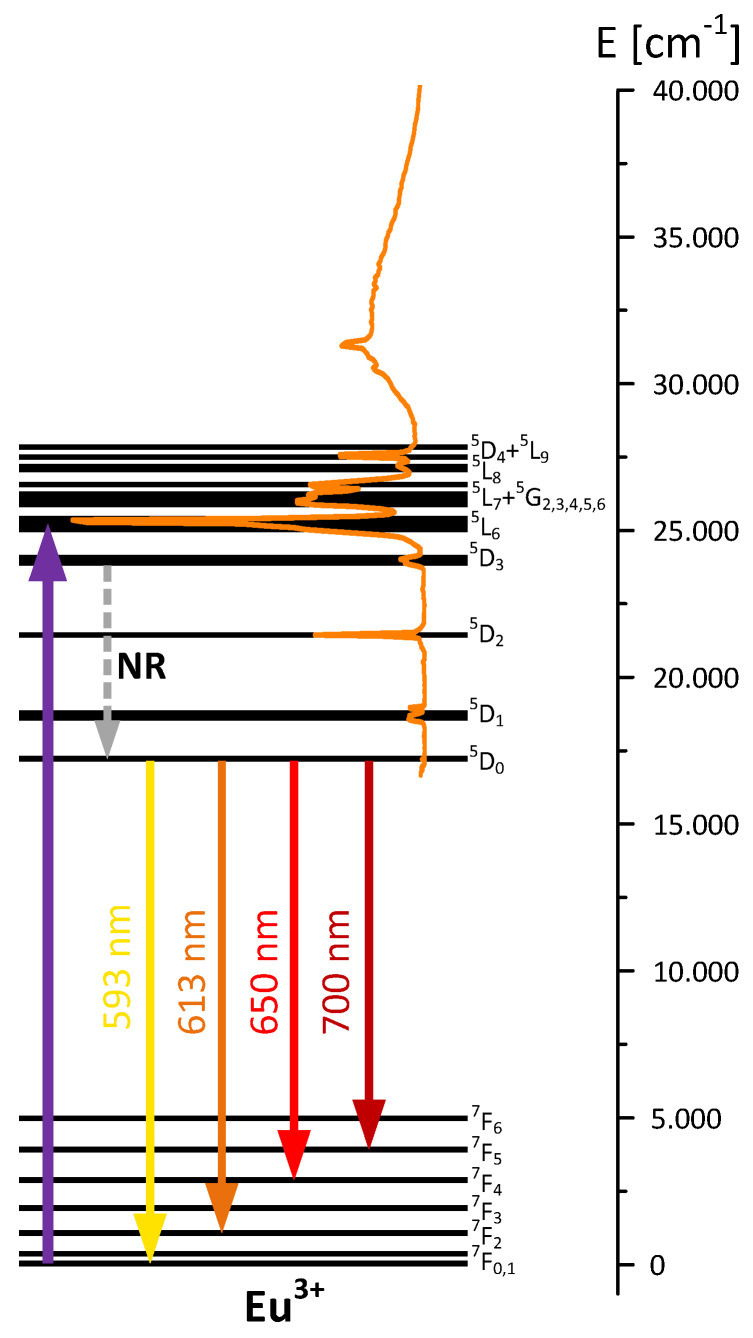
Scheme of the energy levels of Eu(III) together with most efficient excitation path, excitation spectrum, and emission transitions from ^5^D_0_ level (NR—non-radiative transitions).

The luminescence spectra of Eu_2_L_3_ complex and BPA.DA-MMA@2%Eu_2_L_3_ composites (Figure 10b) exhibited behaviors typical of the europium trivalent ion and included several emission lines in the visible range, related to optical transitions from the metastable 5D_0_ singlet to ^7^F_J_ multiplets (shown schematically in Figure 11). The most intense line was centered at 613 nm, corresponding to the ^5^D_0_ → ^7^F_2_ transition, responsible for the typical orange-red luminescence observed in europium-doped phosphors. It should be noted that quite strong broadband luminescence from the polymer host was observed in the hybrid material, significantly affecting the character of the emission spectrum. As in the previous case, the recorded fluorescence decays had the same character for all emission lines. The decay characteristics (presented in the inset of Figure 10b) are nearly exponential with the time constant of the order of 240 µs. For the hybrid material, rapid activity was clearly apparent in the initial part of the decay, related to the polymer host luminescence.

The excitation spectra of the tested lanthanide complexes indicate that the organic ligands used, i.e., 1H-pyrazole-3,5-dicarboxylates, do not show the optical features that would be supportive for the efficient transfer of the energy to lanthanide ions. Therefore, the desired “antenna effect” significantly enhancing the efficiency of luminescence was not confirmed in the investigated materials.

In the case of Tb_2_L_3_ compounds, the band related to excitation by ligand was clearly visible on the excitation spectrum (see figure below), corresponding with energy of ca. 37,000 cm^−1^. However, the efficiency of excitation by ligand was nearly equal to the direct excitation efficiency, meaning that the position of the triplet level does not overlap well with the energy-accepting levels of terbium, and therefore does not support the efficient transfer of energy to the rare-earth ions.

In the case of the Eu_2_L_3_ complex, the excitation spectrum was dominated by f-f transitions of Eu(III) ions, and excitation via ligand hardly possible, which indicates the significant mismatch between the position of the triplet state and the rare-earth ion energy levels. Therefore, the energy transfer from ligand to Ln(III) ions was totally inefficient.

In general, when energy-absorbing states of lanthanide(III) ions are only slightly lower in energy than the triplet state of the ligand, one can expect strong metal-ion fluorescence. Although we were unable to measure the position of the triplet state, it is obvious that in the investigated case this condition was not met. We would guess that in both cases the triplet levels were located too high (30,000 cm^−1^ or above) to provide the efficient energy coupling between ligand and rare earth.

Methods of predicting and tailoring the processes of energy transfer between ligands and rare-earth ions will be the subject of further studies.

It should also be noted that with respect to emission intensity and fluorescence decay profiles, there were no significant differences between powders and bulk hybrid materials. Unfortunately, the emission intensity for all materials was much weaker than for the original lanthanide complexes, so the hybrid materials require further improvement in terms of optical quality and transparency. The fluorescence decay rate remained the same, independently from the concentration of the metal complexes and the form of the samples, indicating that active ions were effectively shielded from the highly energetic phonons of the polymer matrix and the ligand remained unchanged during the processing of the materials.

## 4. Conclusions

A series of novel luminescent hybrid materials have been presented, designed based on the top-down procedure. Coordination polymers of Eu(III) and Tb(III) ions with 1H-pyrazole-3,5-dicarboxylate ligand were incorporated in solid form into the polymeric BPA.DA-MMA matrix as luminescence donates. Material degradation pathways were found to be dominated by components of the polymer matrix and lanthanide(III) complex, as were their forms and heating conditions. The resulting series of hybrid materials demonstrated comparatively good thermal stability in the air atmosphere, but worse in comparison with the matrix. Materials with the addition of the europium complex were more stable than those with the terbium complex. In the inert atmosphere, an increase of thermal stability was observed in all the investigated materials as expected due to the limited oxidizing processes. The pyrolysis of the materials in nitrogen led to the evolution of products including water, carbon oxides, methacrylic acid and its derivatives, as well as bisphenol A and its decomposition compounds. In an air atmosphere, products of BPA decomposition were not observed, while a high intensity of carbon dioxide was apparent as an effect of the oxidation reactions. There were variations in the thermal behaviors of the block and powdered materials. Heating of the block materials in the air also allowed endothermic effects to be distinguished on the DSC curves, while for powdered materials mainly exothermic effects were observed.

All series of hybrid materials doped with different concentrations of europium(III) exhibited characteristic orange-red luminescence, while materials doped with terbium(III) emitted green light. The emission intensity and fluorescence decay profiles were independent of the form of the investigated materials. After improvement of optical properties, the resulting materials may be suitable for curing in thin layers and can be used as special protective coatings on a variety of materials.

## Figures and Tables

**Table 1:** Thermal characterization of matrix and materials (block) based on the TG-DSC curves in air.

	Stage I	Effect I		Effect II		Stage II	Effect III	
	∆T_1_ (°C) ∆m_1_ (%)	T_onset_/T_peak_ (°C)	ΔH_1_ (J/g)	T_onset_/T_peak_ (°C)	∆H_2_ (J/g)	∆T_2_ (°C) ∆m_2_ (%)	T_onset_/T_peak_ (°C)	ΔH_3_ (J/g)
BPA.DA-MMA	229–448 74.8	395.4/401.2	11.3	443/448.4	129.8	449–592 25.2	- -	-
BPA.DA-MMA@Eu_2_L_3_	163–454 73.3	392.4/401.2	512.6	428.3/444.8	77.9	455–586 26.5	- -	-
BPA.DA-MMA@Tb_2_L_3_	119–454 74.1	385.2/389.7	220.3	404.2/414	343.8	455–607 25.6	431.6/440.6	130.1

**Table 2:** Comparison of thermal behavior of investigated materials in the form of block (B) and powder (P) in air atmosphere.

**Mass Loss (%)**	**Temperature (°C) for Materials BPA.DA-MMA@Eu_2_L_3_ with Different Content of Metal Complex (B/P)**				
	**0.1%**	**0.2%**	**0.5%**	**1%**	**2%**
1	198/164	203/165	187/159	190/176	162/159
5	339/303	333/299	327/307	331/304	325/303
20	399/344	390/343	389/347	389/345	387/346
50	423/374	420/373	420/376	420/374	420/375
**Mass Loss (%)**	**Temperature (°C) for Materials BPA.DA-MMA@Tb_2_L_3_ with Different Content of Metal Complex (B/P)**				
	**0.1%**	**0.2%**	**0.5%**	**1%**	**2%**
1	161/153	196/187	207/184	199/149	118/155
5	311/300	332/300	329/303	333/301	315/301
20	379/345	391/345	386/347	393/347	379/341
50	419/373	421/375	421/375	421/378	420/373

## Data Availability

All data are available from the corresponding author upon reasonable request.

## Supplementary Materials

The following supporting information can be downloaded at: https://www.mdpi.com/article/10.3390/ma15248826/s1, Figure S1: ATR-FTIR spectra of BPA.DA-MMA (polymeric matrix) and BPA.DA-MMA@0.1–2%Eu_2_L_3_; Figure S2: ATR-FTIR spectra of BPA.DA-MMA (polymeric matrix) and BPA.DA-MMA@0.1–2%Tb_2_L_3_; Figure S3: The transmittance FTIR spectra; Figure S4: The TG curves of powder and block hybrid materials doped with europium complex (air); Figure S5: TG/DTG/DSC curves of polymeric matrix; Figure S6: TG/DTG/DSC curves of hybrid materials doped with europium(III) complex; Figure S7: TG/DTG/DSC curves of hybrid materials doped with terbium(III) complex; Figure S8: TG/DTG/DSC curves of doped complexes: (**a**) hexahydrate europium complex and (**b**) hexahydrate terbium complex (air); Figure S9: Gram–Schmidt plots; Figure S10: Comparison of gas (air/nitrogen) products at time of 19.750 min; Table S1: Parameters of materials synthesis; Table S2: Thermogravimetric results of free matrix and hybrid materials with 2 wt.% dopant (blocks) in air and nitrogen.
