# Supplementary material for: A Top-Down Approach and Thermal Characterization of Luminescent Hybrid BPA.DA-MMA@Ln2L3 Materials Based on Lanthanide(III) 1H-Pyrazole-3,5-Dicarboxylates
Source: Materials (Basel). 2022 Dec 10;15(24):8826. doi: 10.3390/ma15248826 (PMC9786596; doi:10.3390/ma15248826)
Supplement: Supplementary file 1 [file materials-15-08826-s001.zip › materials-1995428-supplementary.pdf]

Table S1. Parameters of materials synthesis.

Material	Eu_2L_3 (g)	Tb_2L_3 (g)	BPA.DA (g)	MMA (g)	IRGACORE (g)
BPA.DA-MMA	-	-	5.6	2.4	0.08
BPA.DA-MMA@0.1% Eu_2L_3	0.008	-	5.6	2.4	0.08
BPA.DA-MMA@0.2% Eu_2L_3	0.016	-	5.6	2.4	0.08
BPA.DA-MMA@0.5% Eu_2L_3	0.04	-	5.6	2.4	0.08
BPA.DA-MMA@1% Eu_2L_3	0.08	-	5.6	2.4	0.08
BPA.DA-MMA@2% Eu_2L_3	0.16	-	5.6	2.4	0.08
BPA.DA-MMA@0.1% Tb_2L_3	-	0.008	5.6	2.4	0.08
BPA.DA-MMA@0.2% Tb_2L_3	-	0.016	5.6	2.4	0.08
BPA.DA-MMA@0.5% Tb_2L_3	-	0.04	5.6	2.4	0.08
BPA.DA-MMA@1% Tb_2L_3	-	0.08	5.6	2.4	0.08
BPA.DA-MMA@2% Tb_2L_3	-	0.16	5.6	2.4	0.08

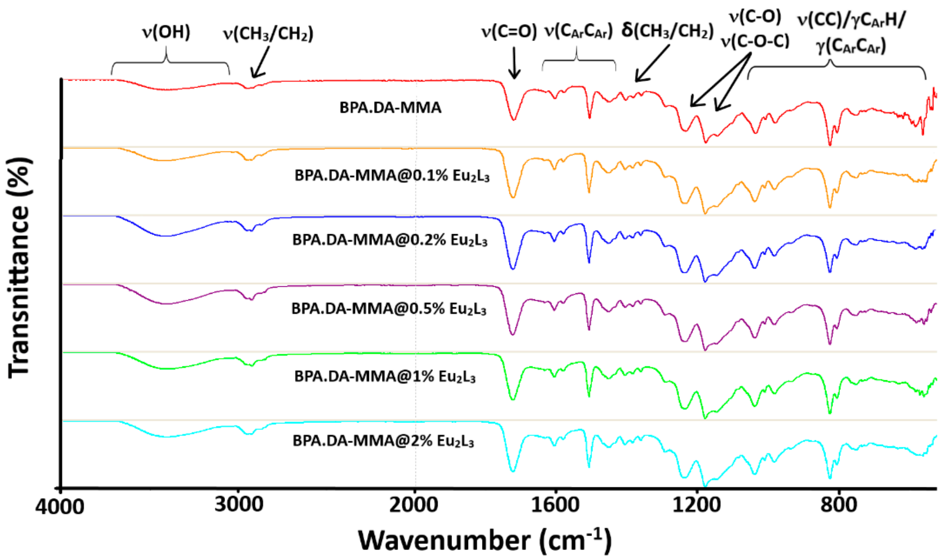

Figure S1. ATR-FTIR spectra of BPA.DA-MMA (polymeric matrix) and BPA.DA-MMA@0.1-2%Eu_2L_3

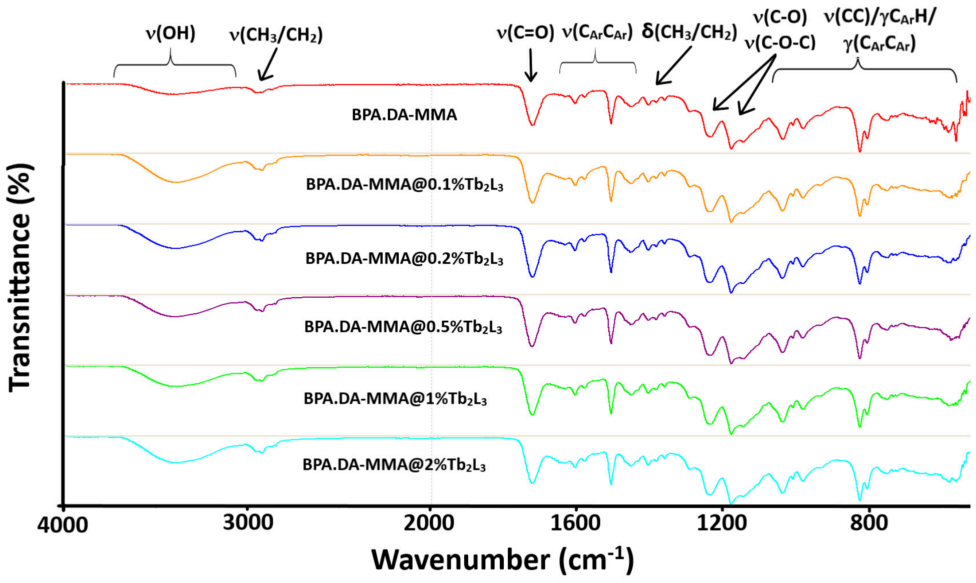

Figure S2. ATR-FTIR spectra of BPA.DA-MMA (polymeric matrix) and BPA.DA-MMA@0.1-2%Tb_2L_3

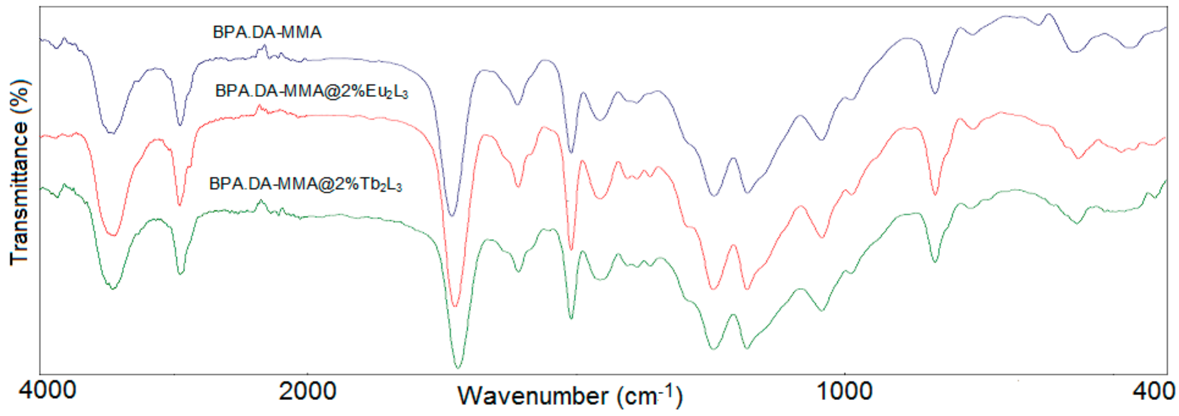

Figure S3. The transmittance FTIR spectra.

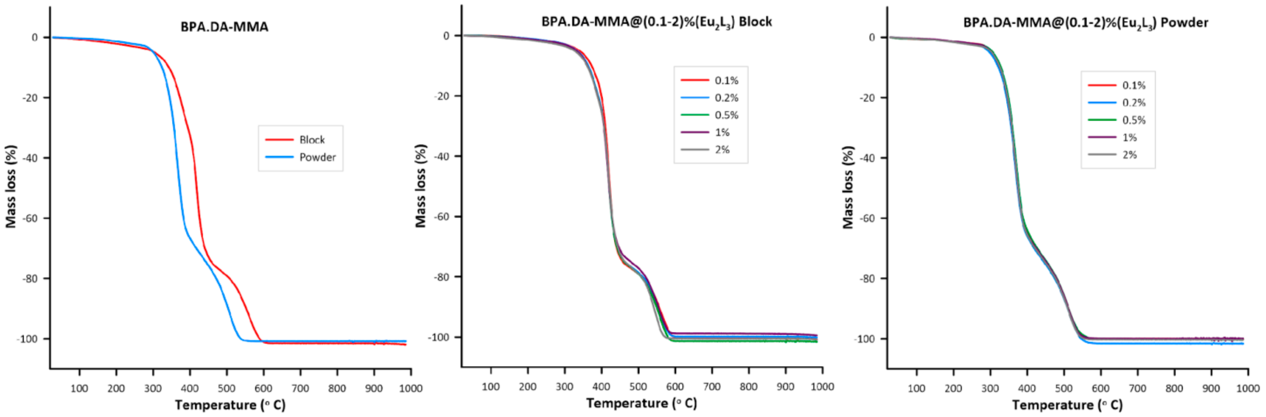

Figure S4. The TG curves of powder and block hybrid materials doped with europium complex (air).

Powder **Block**

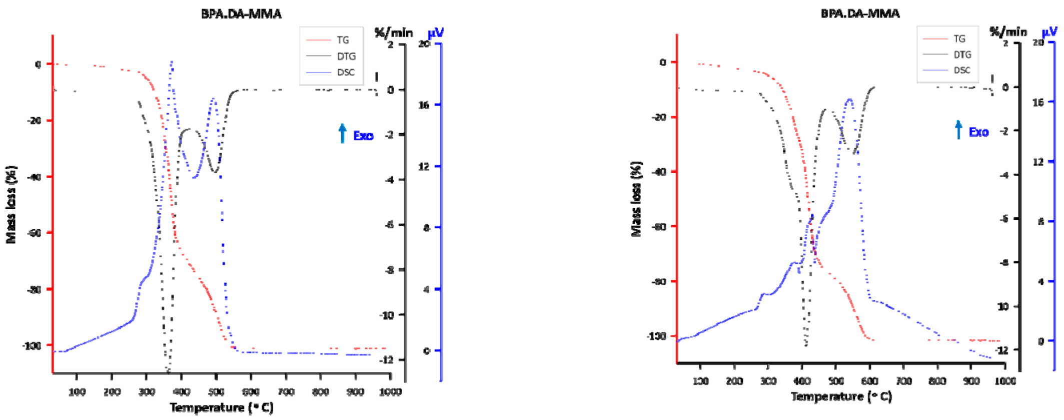

Figure S5. TG/DTG/DSC curves of polymeric matrix.

Powder

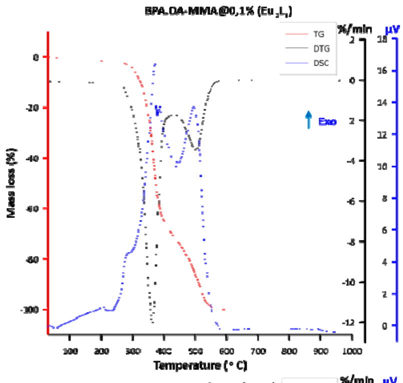

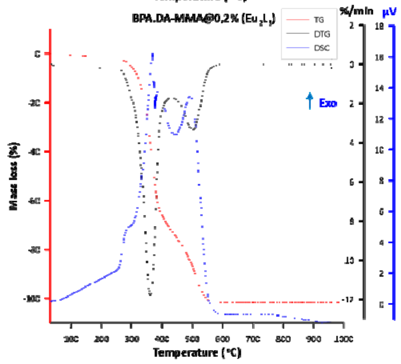

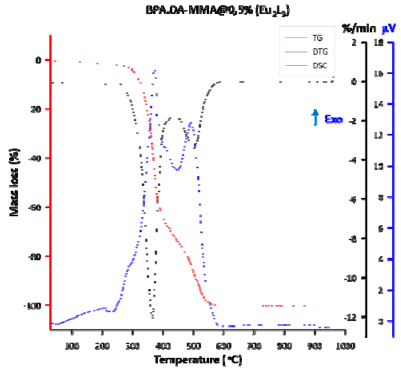

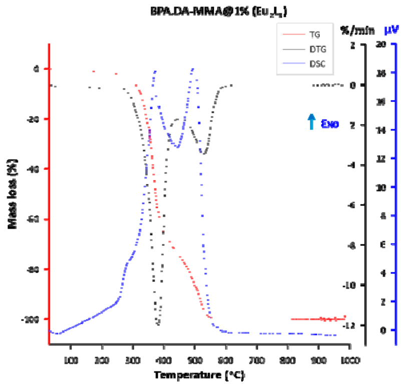

Block

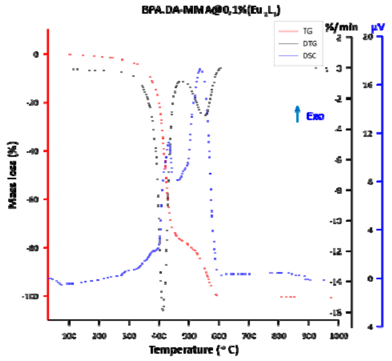

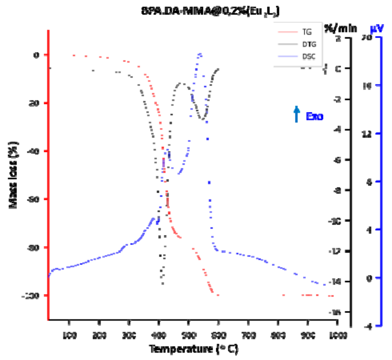

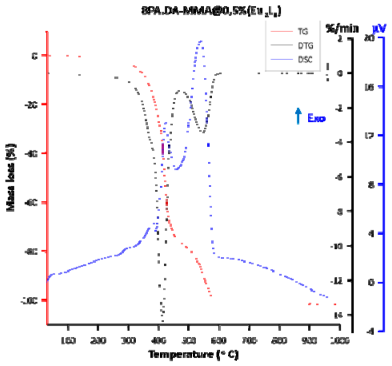

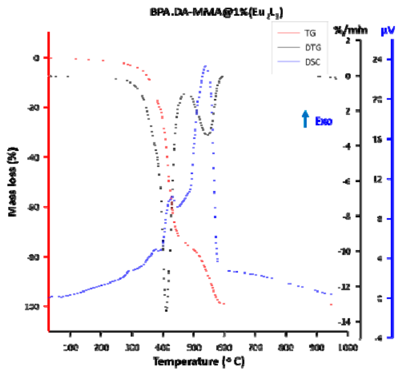

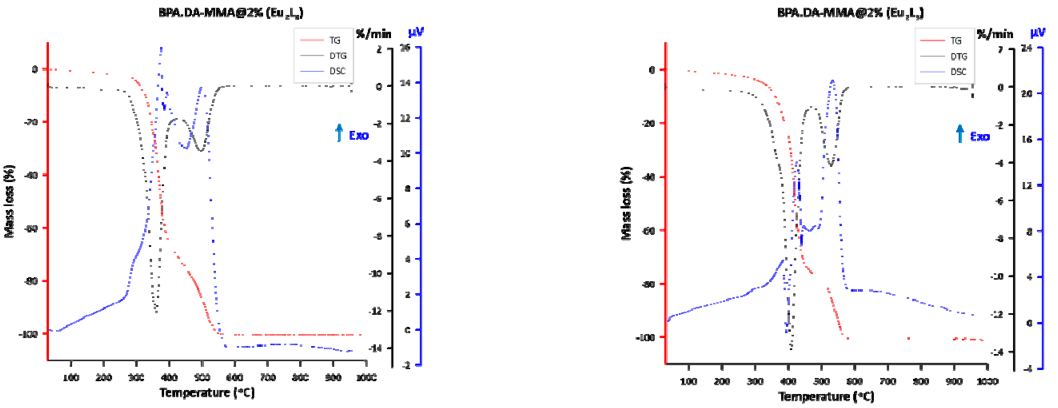

Figure S6. TG/DTG/DSC curves of hybrid materials doped with europium(III) complex.

Powder

Block

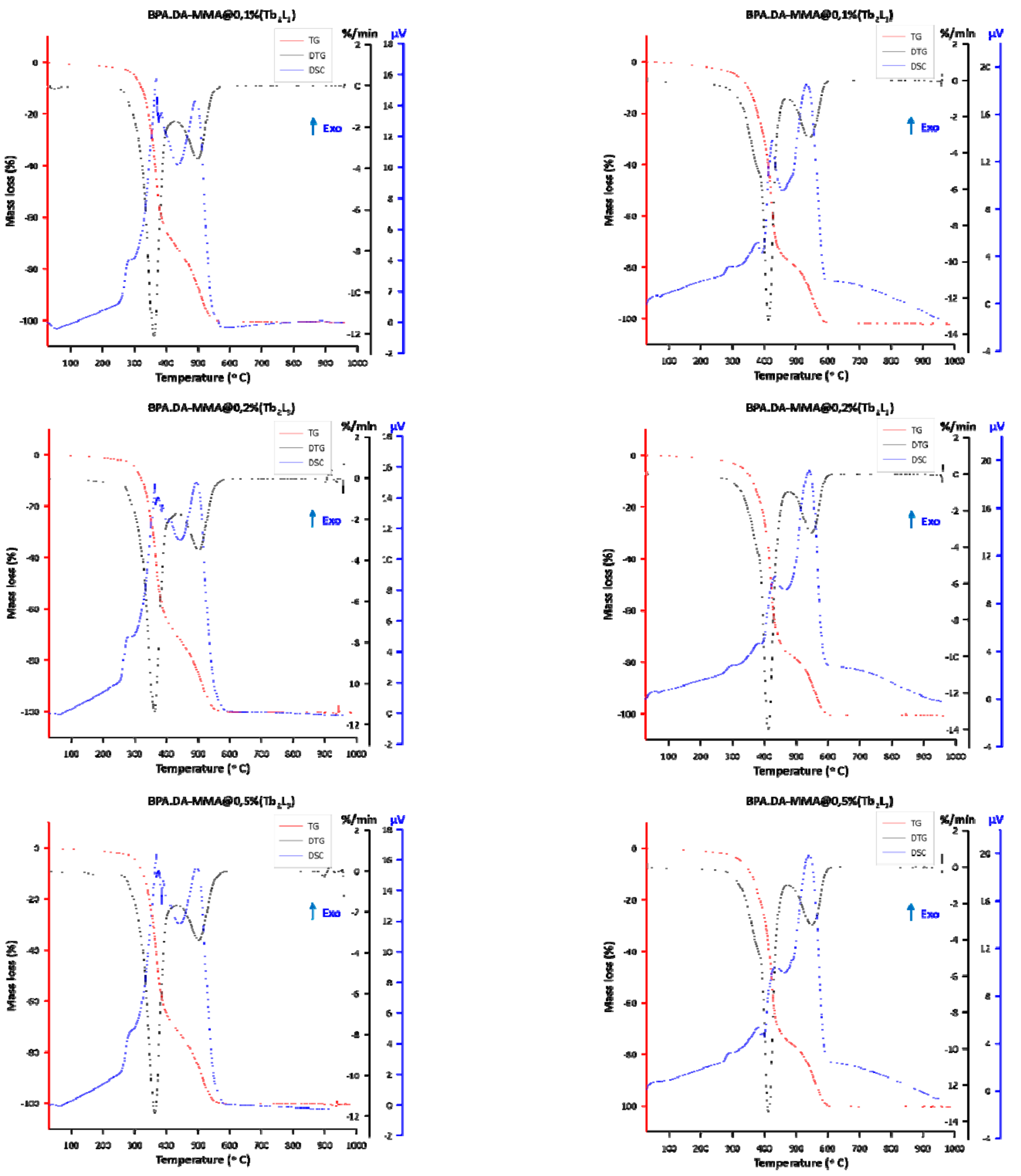

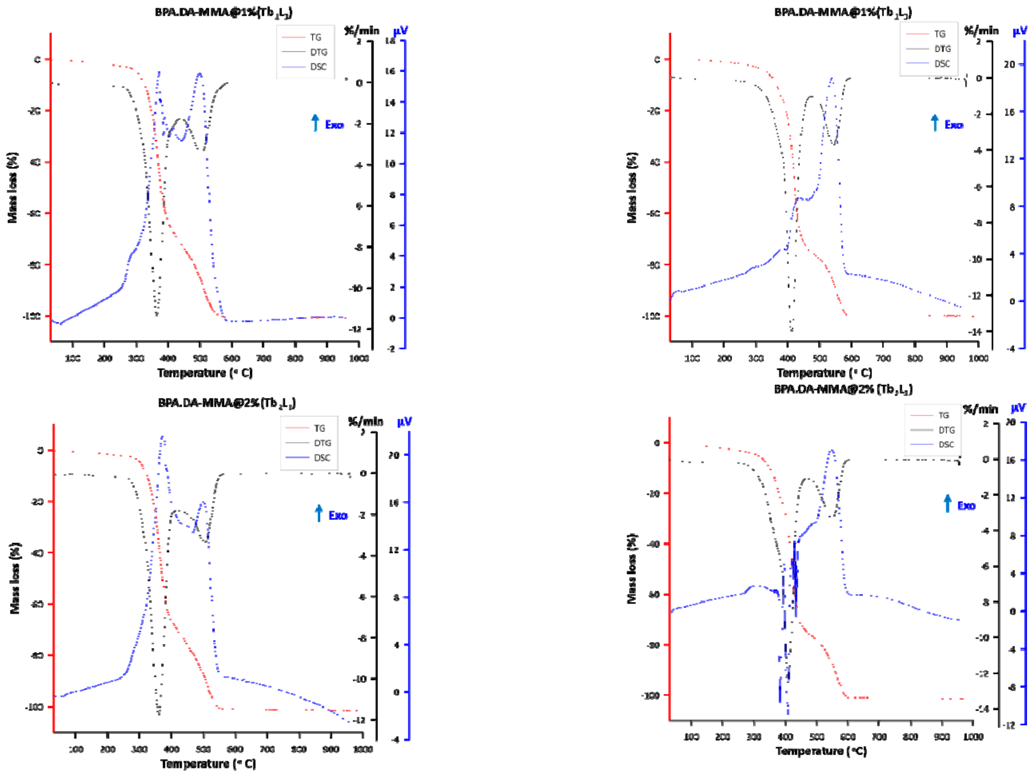

Figure S7. TG/DTG/DSC curves of hybrid materials doped with terbium(III) complex.

Table S2. Thermogravimetric results of free matrix and hybrid materials with 2 wt. % amount of dopant (blocks) in air and nitrogen.

Compounds	Mass loss	Temperature (ºC) air	Temperature (ºC) nitrogen
BPA.DA-MMA	1%	228	261
	5%	340	352
	20%	399	397
	50%	420	419
BPA.DA-MMA@2% Eu_2L_3	1%	162	264
	5%	325	361
	20%	387	400
	50%	420	421
BPA.DA-MMA@2% Tb_2L_3	1%	118	267
	5%	315	361
	20%	379	401
	50%	420	424

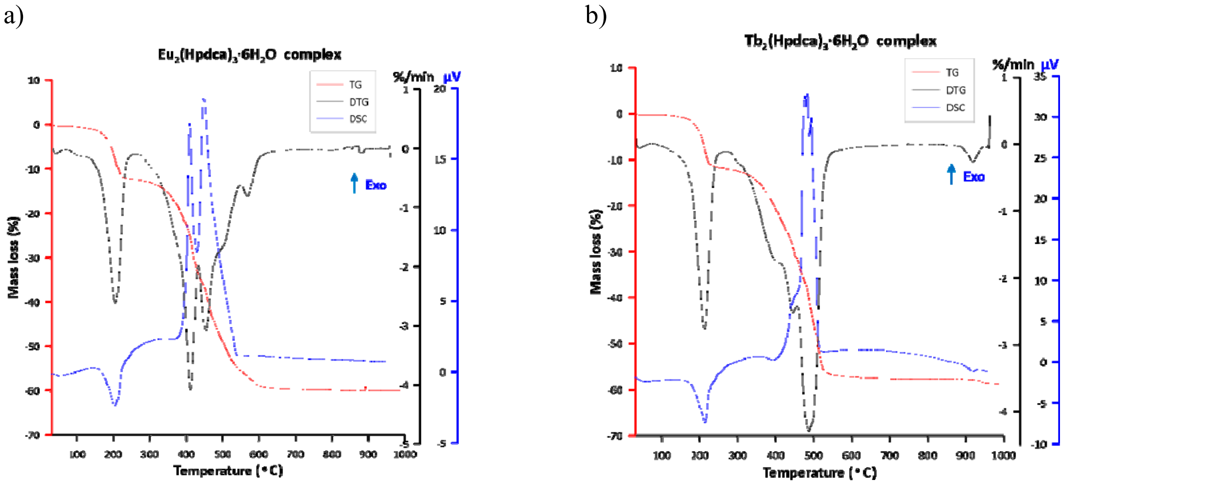

Figure S8. TG/DTG/DSC curves doped complexes: a) hexahydrate europium complex and b) hexahydrate terbium complex (air).

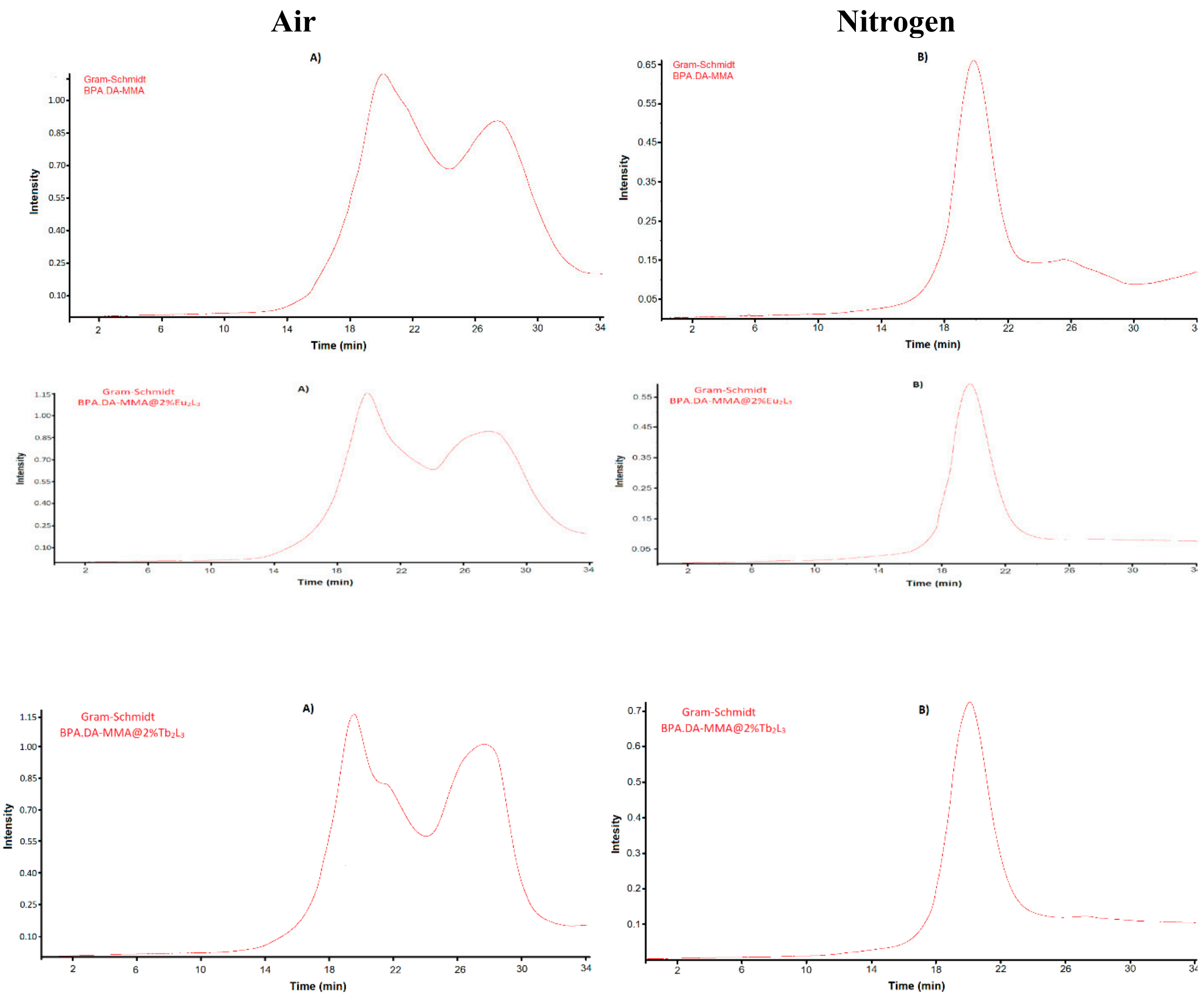

Figure S9. Gram-Schmidt plots.

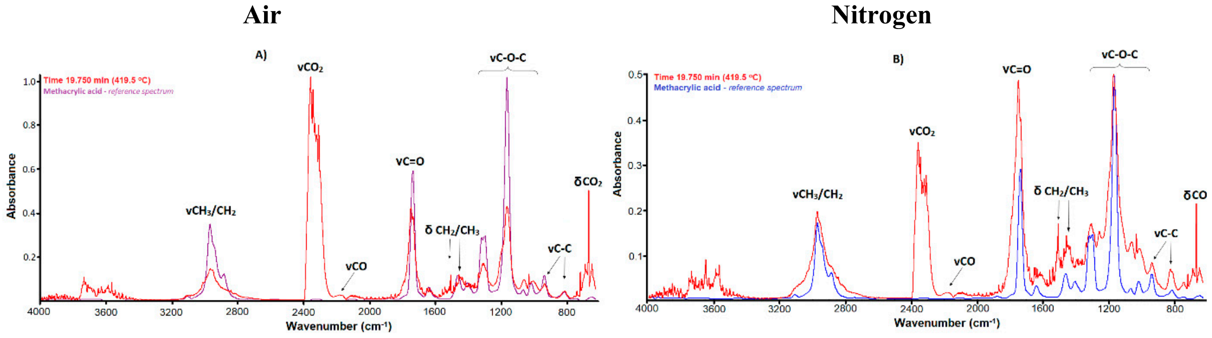

Figure S10. Compare gas (air/nitrogen) products in the time: 19.750 min.
